# Exome sequencing of osteosarcoma reveals mutation signatures reminiscent of BRCA deficiency

**DOI:** 10.1038/ncomms9940

**Published:** 2015-12-03

**Authors:** Michal Kovac, Claudia Blattmann, Sebastian Ribi, Jan Smida, Nikola S. Mueller, Florian Engert, Francesc Castro-Giner, Joachim Weischenfeldt, Monika Kovacova, Andreas Krieg, Dimosthenis Andreou, Per-Ulf Tunn, Hans Roland Dürr, Hans Rechl, Klaus-Dieter Schaser, Ingo Melcher, Stefan Burdach, Andreas Kulozik, Katja Specht, Karl Heinimann, Simone Fulda, Stefan Bielack, Gernot Jundt, Ian Tomlinson, Jan O. Korbel, Michaela Nathrath, Daniel Baumhoer

**Affiliations:** 1 Bone Tumour Reference Center at the Institute of Pathology, University Hospital Basel, Schönbeinstrasse 40, 4031 Basel, Switzerland; 2 The Wellcome Trust Centre for Human Genetics, University of Oxford, Roosevelt Drive, Oxford OX3 7BN, UK; 3 Pediatrics 5 (Oncology, Hematology, Immunology), Klinikum Stuttgart Olgahospital, Kriegsbergstrasse 62, 70174 Stuttgart, Germany; 4 Department of Pediatric Hematology, Oncology, Immunology and Pulmology, University of Heidelberg, Im Neuenheimer Feld 430, 69120 Heidelberg, Germany; 5 Institute of Radiation Biology, Clinical Cooperation Group Osteosarcoma, Helmholtz Zentrum München, Ingolstädter Landstrasse 1, 85764 Neuherberg, Germany; 6 Pediatric Oncology Center, Department of Pediatrics, Technische Universität München and Comprehensive Cancer Center, Kölner Platz 1, 80804 Munich, Germany; 7 Institute of Computational Biology, Helmholtz Zentrum München, Ingolstädter Landstrasse 1, 85764 Neuherberg, Germany; 8 Institute for Experimental Cancer Research in Pediatrics, Goethe-University, Komturstrasse 3a, 60528 Frankfurt, Germany; 9 German Cancer Consortium (DKTK), Im Neuenheimer Feld 280, 69120 Heidelberg, Germany; 10 German Cancer Research Center (DKFZ), Im Neuenheimer Feld 280, 69120 Heidelberg, Germany; 11 European Molecular Biology Laboratory (EMBL), Genome Biology Unit, Meyerhofstrasse 1, 69117 Heidelberg, Germany; 12 The Institute of Mathematics and Physics, Faculty of Mechanical Engineering, Slovak University of Technology, 84248 Bratislava, Slovak Republic; 13 Orthopaedic Department, Basel University Childrens Hospital (UKBB), Spitalstrasse 33, 4056 Basel, Switzerland; 14 Department of Orthopedic Oncology, Sarcoma Center Berlin-Brandenburg, HELIOS Klinikum Berlin-Buch, Schwanebecker Chaussee 50, 13125 Berlin, Germany; 15 Department of Orthopedic Surgery, Ludwig-Maximilians-University Munich, Campus Grosshadern, Marchionistrasse 15, 81377 Munich, Germany; 16 Clinic and Policlinic of Orthopedics and Sports Orthopedics, Technische Universität München, Ismaninger Strasse 22, 81675 Munich, Germany; 17 Department of Orthopaedics and Trauma Surgery, University Hospital Dresden, Fetscherstrasse 74, 01307 Dresden, Germany; 18 Center for Musculoskeletal Surgery, Charité—University Medicine Berlin, Campus Virchow Klinikum, Augustenburger Platz 1, 13353 Berlin, Germany; 19 Institute of Pathology, Technische Universität München, Trogerstrasse 18, 81675 Munich, Germany; 20 Medical Genetics, University Hospital Basel, Burgfelderstrasse 101, 4055 Basel, Switzerland; 21 Department of Pediatric Oncology, Klinikum Kassel, Mönchebergstrasse 41-43, 34125 Kassel, Germany

## Abstract

Osteosarcomas are aggressive bone tumours with a high degree of genetic heterogeneity, which has historically complicated driver gene discovery. Here we sequence exomes of 31 tumours and decipher their evolutionary landscape by inferring clonality of the individual mutation events. Exome findings are interpreted in the context of mutation and SNP array data from a replication set of 92 tumours. We identify 14 genes as the main drivers, of which some were formerly unknown in the context of osteosarcoma. None of the drivers is clearly responsible for the majority of tumours and even *TP53* mutations are frequently mapped into subclones. However, >80% of osteosarcomas exhibit a specific combination of single-base substitutions, LOH, or large-scale genome instability signatures characteristic of BRCA1/2-deficient tumours. Our findings imply that multiple oncogenic pathways drive chromosomal instability during osteosarcoma evolution and result in the acquisition of BRCA-like traits, which could be therapeutically exploited.

## Abstract (web summary)

Osteosarcomas are a heterogenous group of tumours and little is known about how these tumours evolve. Here, Kovac *et al*. use exome sequencing and discover that although no driver gene explains the majority of these tumours, they are characterized by specific mutation signatures and genomic instability typical of BRCA1/2-deficient tumours.

## Graphical abstract

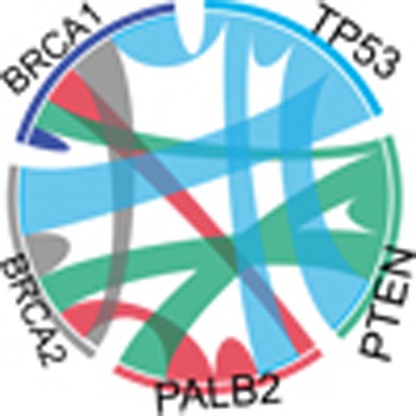

Osteosarcomas (OS) are primary malignant tumours of bone with complex karyotypes showing abundant structural and numerical aberrations. Rapid tumour progression and early metastatic spread are the rational for multimodal treatment approaches that can achieve long-term survival in about 60% of patients. Effective treatment options are still lacking for the remaining 40% of patients suffering from refractory or recurrent disease, however^1^. A partial explanation for treatment failure might lie in different aetiologies responsible for the structural aberrations marking the onset of the disease and resulting in a variety of mutations in genes and pathways of which only few are targetable. OS arise owing to mutations in the *TP53* tumour suppressor gene^2^,^3^,^4^ and a plethora of other cancer drivers, as for example *RB1* (refs 2, 3, 4), *ATRX*^2^,^3^, *DLG23* (ref. 2), *RUNX2* (ref. 5), *WRN*^6^,^7^, *RECQL4* (refs 5, 7, 8), *CDKN2A/B*^9^, *BLM*^7^, *PTEN*^3^ and other PI3K/Akt/mTOR pathway members‘ each of which contribute only to a small proportion of patients. As a consequence, widely applicable therapeutic targets have not been identified so far.

The introduction of high-throughput sequencing of cancer genomes has expanded a list of potential OS driver genes, but disappointingly failed to provide important leads that could improve patients’s care. In fact, data published so far only confirmed that the intricate nature of these tumours develops through cancer heterogeneity promoting the accumulation of chromosomal aberrations, kataegis and chromothripsis^2^,^3^,^4^. Marked inter- and intratumoural heterogeneity seems to be linked to the observed differences in treatment efficacy and clinical outcome of the respective patients^10^, but the particular sets of mutations underlying these patterns, the clonal frequencies of driver mutations and how different (sub-)populations of tumour cells relate functionally to each other, remains largely unknown. In this study, we search for driver gene mutations in 123 OS and attempt to reduce the complexity of their genomes by applying abstraction analyses supplemented by clonal mapping and ordering. Finding common traits of different cell lineages within and between tumours seems to constitute a first step towards developing more individualized and effective treatment strategies in the future.

## Results

### Mutation processes moulding the exomes of 31 OS

**Figure 1:**
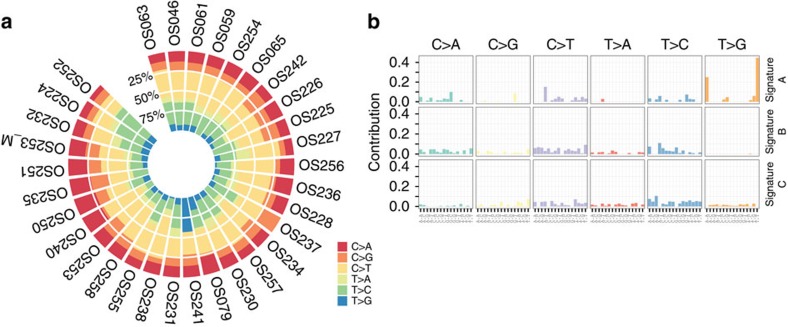
Somatic SNV spectra and mutation signatures. (**a**,**b**) Data are derived from exomes of each tumour. Note that OS with very few somatic SNVs are included for sake of completeness.

Our discovery set for whole-exome sequencing comprised 31 OS, which were sequenced at a median depth of ∼150 × (range 131–370) alongside with paired constitutional DNA from peripheral blood (Table 1). All tumour samples were derived from pre-therapeutic biopsies. Making use of Stampy and Platypus programs for mapping and variant calling, the number of somatic base substitutions (single-nucleotide variants (SNVs)) and indels called with high confidence ranged from 7 to 3,153 (median=66.5) and 17 to 529 (median=33.5) per exome, respectively. Of these, a median of 21 SNV changes (range 4–174) and seven small indels (range 2–210) were potentially functional within protein-coding regions. The SNV spectrum for each OS exome is shown in Fig. 1a and Supplementary Figs 1 and 2. With two exceptions, the C:G>T:A changes were the most common changes followed by C:G>A:T and T:A>C:G changes. The mutation burden of OS was similar to the ageing signature of most tumour types from the pan-cancer analysis^11^, however, a decomposition of mutation spectra with a non-negative matrix factorization algorithm revealed a pattern similar to the signatures 3 and 5 (Fig. 1b and Supplementary Fig. 3). A subset of tumours of this class is known to acquire a characteristic pattern of substitution mutations including kataegis and the presence of signature 3 was strongly associated with *BRCA1* and *BRCA2* mutations within breast and pancreatic cancer types^11^,^12^.

### Driver mutations impair cell control of genome integrity

For the discovery of somatic driver mutations we restricted our analysis to protein-coding regions. A minimum variant allele frequency of 0.05 was set. We then prioritized genes for further investigation by filtering mutations to exclude all SNVs with moderate or benign predicted functional effects (Sorting Intolerant From Tolerant (SIFT) score=<0.05 and Mutation Taster and PolyPhen2 >0.8). All protein-truncating and splice-site mutations were retained. We then identified genes that were mutated in two or more tumours and inspected all variant reads in the Integrated Genome Viewer to exclude any mutations at sites of evidently poor quality. Eventually, 26 genes (Supplementary Data 1) including some that had already been reported in the context of OS (for example, *ARTX*, *SFPQ*, *FGFRL1* and *RB1*) remained^2^,^3^,^4^,^13^.

Since patients with Werner, Li–Fraumeni, Rothmund–Thomson and Bloom syndromes have a comparably higher risk of OS^8^,^14^,^15^, we examined our sequencing data for germline mutations in the *WRN*, *TP53*, *RECQL4* and *BLM* genes. We identified two germline *TP53* mutations (OS-241 and OS-228), one germline *WRN* mutation in addition to loss of heterozygosity (LOH) around the locus (OS-230) and two rare germline *RECQL4* variants with unknown significance (OS-227 and OS-238). We then applied the same prioritization scheme to the remainder of genes with germline variants. Prioritized cancer drivers not previously reported in OS included *RET*, *MUTYH*, *NUMA1*, *FANCA*, *BRCA2* and *ATM*. We chose these genes for further investigation, based on the presence of at least two clearly pathogenic mutations and/or co-segregation of mutations with cancer in the respective families. Furthermore, these genes were excellent functional candidates.

We validated mutations detected in 388 genes (Supplementary Data 2) by Ion Torrent sequencing in the discovery set of tumours and then undertook replication testing of 30 genes (Supplementary Data 3) in a set of additional 92 unpaired OS. The analysis of merged variant calls from 123 tumours yielded a total of 22, 10 and 11 *TP53*, *RB1* and *ATRX* mutations, respectively (Supplementary Table 1). Three tumours acquired somatic *RET* mutations. One patient, OS-250, carried a germline *RET* mutation, which at the time of diagnosis had not yet manifested by multiple endocrine neoplasia type 2 but the mutation co-segregated with breast cancer and rhabdomyosarcoma in two first-degree relatives. Two *de novo* germline *RET* mutations previously associated with late-onset multiple endocrine neoplasia type 2 were identified in patients OS-224 and OS-242. We also identified seven *MUTYH* mutations (germline or somatic) and four, four, three and one mutations in the *FANCA*, *MDC1*, *NUMA1* and *PTEN* genes, respectively. Somatic missense mutations in *MDC1* and *NUMA1* genes affected conserved residues of protein domains encoding nuclear localization signal and/or interacting with other proteins of BRCA complex, for example CHEK2. *MUTYH* mutations were equally distributed between an endonuclease domain and a NUDIX-type hydrolase domain. There were also two somatic *WRN* mutations affecting a DNA-binding domain and three germline mutations in the *ATM* gene, two of which have been linked to breast cancer susceptibility before^16^,^17^.

The Intogen^18^ pathway analysis reassuringly identified *ATRX* and *RB1* as the main drivers (Supplementary Data 4). The Intogen list of drivers did not include *TP53* since there was only one mutation in the discovery set of tumours, but *TP53* and three other genes (*RB1*, *ATRX* and *ATM*) were notably reported by Intogen as drivers in the pan-cancer analysis^19^. Exploring gene ontology of the remaining genes we found that many, if not all genes, have been functionally related to DNA damage repair, chromosomal segregation and cell cycle control of genome integrity^8^,^20^,^21^,^22^,^23^,^24^.

### Structural and copy-number alterations

**Figure 2:**
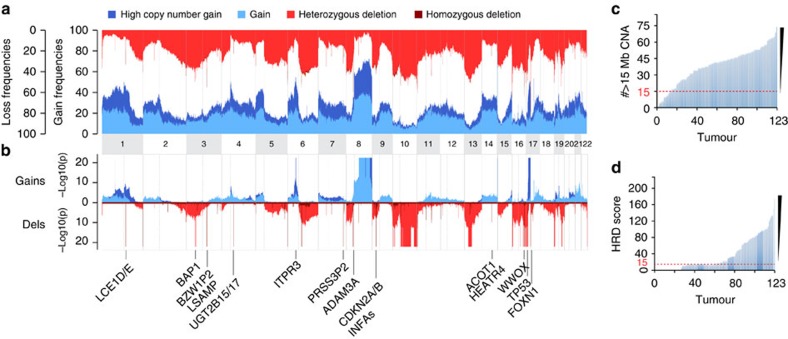
Copy-number variation analysis. (**a**) Copy-number profile of 123 OS genomes. (**b**) Significantly over-represented copy-number alterations. The most important over-represented SCNA loci are highlighted. Blue, gains; red, losses. (**c**) The number of copy-number alterations fulfilling specific criteria of a BRCA-like phenotype shown for each tumour (threshold line in red). (**d**) Homologous recombination deficiency (HRD) score shown for each tumour. The threshold for considering a tumour to be BRCA deficient was set to 15 (red dashed line).

We next turned our attention to somatic copy-number alterations (SCNA; Fig. 2a,b), which affected 0.2 (OS-251) to 87% (OS-038) of OS genomes. The median size of a SCNA called with high-sensitivity settings was 4.7 Mb and a typical genome contained 69 of such events. Analysis of single-nucleotide polymorphism arrays using dedicated data-processing methods identified large-scale genomic instability^25^ and LOH^26^,^27^ signatures similar to that of breast and ovarian cancers with BRCA1/2 inactivation in 91% (112/123; Fig. 2c) and 78% (96/123; Fig. 2d) of OS, respectively.

The most frequent SCNA events involved gains of the long arm of chromosome 8 (75%). It has been plausibly suggested that gains of 8q target *MYC* in OS, but chromosome 8 gains in our set of tumours involved very long segments without evidence of targeting any gene specifically. Similarly, other frequent gains of 1p (55%), 1q (53%), 5p (46%), 6p (56%), 17p (66%) and 18p (33%) involved large regions. Large deletions were almost as common as gains, the most frequent involving chromosomes 3 (50%), 6q (45%), 5q (40%), 8p (43%), 10 (56%), 13 (50%), 16 (64%), 17 (47%), 18q (34%) and 19 (54%). Deletions of these chromosomes almost invariably included main OS drivers including *TP53*, *BRCA2*, *RET*, *WRN*, *FANCA* and *RB1* (Fisher exact test, *P*<0.01; Supplementary Fig. 4). We also found good evidence supporting SCNA mutations in the *BRCA1* gene itself (26%) and in members of the homologous recombination repair pathway—*BAP1* (38%), *PTEN* (50%) and *PALB2* (43%)—in which exome sequencing did not identify any mutation.

We specifically searched for small (<1 Mb) and focal SNCAs that might represent oncogene amplifications or tumour suppressor deletions. After filtering out common variants, 20,758 regions were identified although only 80 were found to be recurrent (defined as having frequencies >15%; Supplementary Fig. 5 and Supplementary Data 5). Two focal SCNA involved gene loci with a strong *a priori* importance in OS^2^,^9^, including a deletion of *CDKN2A/2B* located at chromosome 9p21 (15%) and a deletion of *DLG2* at chromosome 11q14 (24%). Other SCNA were detected in known fragile sites (WWOX, for example), deeply intragenic regions as well as microRNAs and genes with no prior association to cancer.

We then assessed the significance of called SCNA regions by using a random sampling model similar to the GISTIC analysis (Supplementary Figs 6 and 7). After filtering for SCNAs with adjusted *P* values <0.01, 92, 412 and 322 amplifications (copy number >4), gains (copy number=3) and deletions (copy number <2) remained (Supplementary Data 6), respectively. *TP53*, *RB1*, *PTEN*, *RUNX2*, *BAP1* and *CDKN2A/B* genes were reassuringly among the over-represented SCNA regions, as well as several new loci including a homozygous loss of interferon genes on chromosome 9 (chr9: 21,248,029–21,408,261) and an amplification of a 10-gene locus on chromosome 1 (chr1: 59,762,489–62,907,413). We then re-examined each candidate SCNA events for association with overall and/or disease-free survival using the Cox regression model. Five loci (including *CDKN2A/B* and *TP53*) were associated with an adverse outcome of patients (Supplementary Fig. 8). Three SCNA loci were associated with earlier onset of disease (Supplementary Fig. 9).

### BRCAness as an emergent property of OS

**Figure 3:**
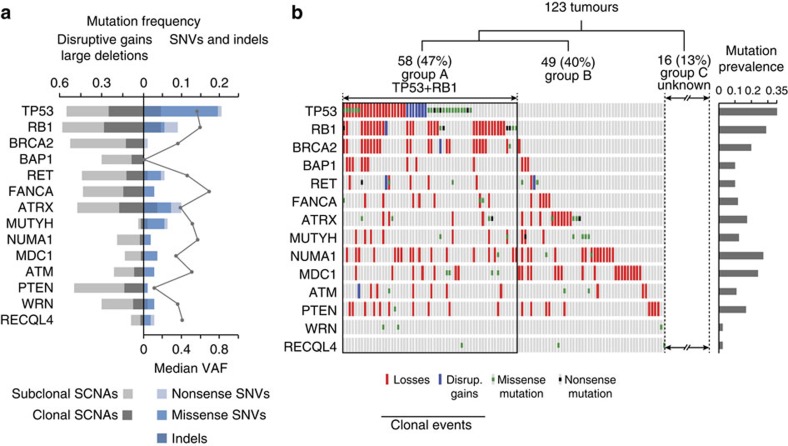
Mutations burden of OS driver genes. (**a**) Mutation profiles of selected OS driver genes, split by copy-number alterations and single-nucleotide changes/indels. The median variant allelic frequency (VAF) is plotted for each gene. (**b**) Distribution of selected somatic SNVs with predicted pathogenic effects and indels across cancers. Note that only clonal copy-number losses and disruptive gains are shown here. Blue, disruptive gains (that is, one breakpoint within a gene); red, losses.

**Figure 4:**
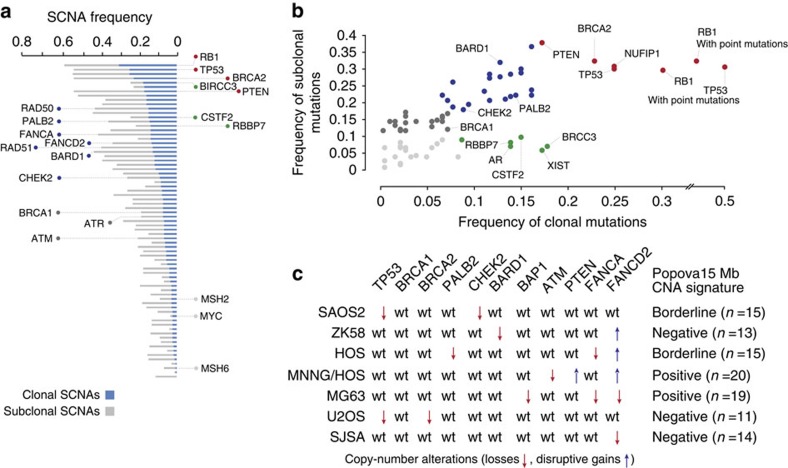
Copy-number alterations in BRCA1/2 and binding partners. (**a**,**b**) Frequencies of clonal and subclonal SCNA mutations. (**c**) SCNA mutational profiles of selected OS cell lines.

Our hypothesis of ‘BRCAness’ does not replace the existing view of OS as a monoclonal expansion of one initial *TP53* mutant cell, but rather offers an explanation of how the vulnerability to chromosomal breakage may be sustained parallel to or in the absence of *TP53* mutations. Using PyClone^28^ estimates of cellular frequencies we found that *TP53* and *RB1* driver mutations were clonal events in 58 tumours (47%; Fig. 3). The remaining tumours (*n*=65, 53%) showed good evidence of the acquisition of genomic instability through alternative pathways—for example, by *MUTYH* mutations^20^—well before *TP53*, *RB1* mutations and/or BRCA-like traits emerged. We envision that OS at this stage also acquired a propensity to shatter into subpopulations of cancer cells. Given enough time, different cancer cell lineages are likely to acquire private SCNAs in the *BRCA1/2* genes (and in their 67 binding partners) simply by chance, and by the time the disease emerges, different clonal populations will have already gained partial or full deficiency in homologous recombination repair. We find this view consistent with evidence that, on average, a typical OS carries 17 SCNA mutations in *BRCA* genes and their core binding partners (Fig. 4a and Supplementary Data 7), mutations in different ‘BRCA’ genes can be functionally equivalent (for example, *PALB2*, *CHEK2*, *PTEN* and *ATM* mutations result in chromosomal instability analogous to BRCA1/2 mutations^29^,^30^,^31^,^32^,^33^) and OS are polyclonal (Table 1).

We therefore estimated the relative importance of each of the 69 ‘BRCA’ genes from the clonality of their SCNA mutations. For Fig. 4b we used a clustering method based on Euclidian distances; the individual position of each gene in the plot is the product of clonal and subclonal frequencies of its SCNA events. It is not surprising that the most frequently mutated and thus outlying genes were *TP53*, *RB1*, *BRCA2* and *PTEN*, and that *TP53* and *RB1* became even more outlying when SNV changes were included. In fact, *TP53* and *RB1* were the only two genes mutated with somatic SNV/indel mutations at a frequency >3%. Putative cancer drivers with a propensity to carry clonal SCNAs were an android receptor gene, the RNA gene *XIST*, the BRCA1 binding partners *RBBP7* and *NCOA2*, a BRCC3 metaloprotease unit of the BRISC complex and CSTF2 that prevents inappropriate RNA processing at sites of DNA repair.

### Efficacy of talazoparib inhibition in OS cell lines

**Figure 5:**
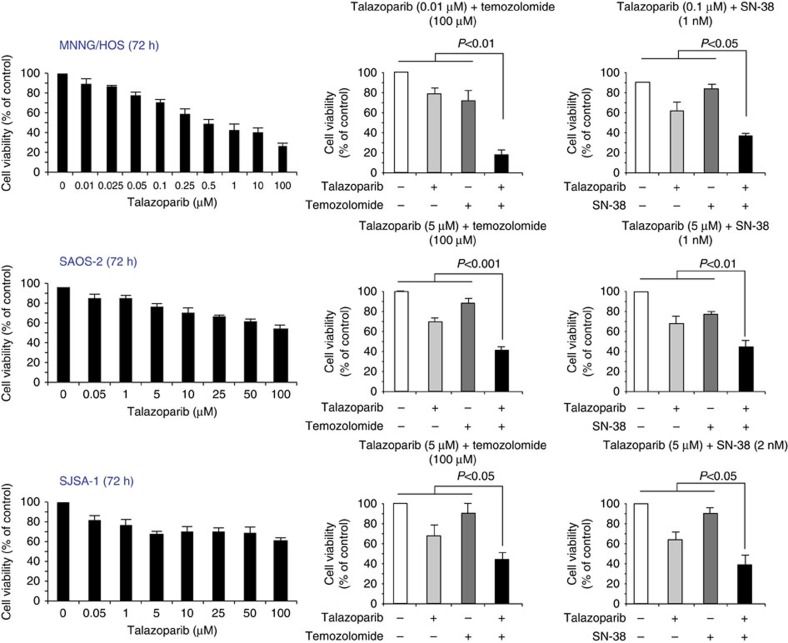
Cell viability assays. MNNG, SAOS2 and SJSA-1 OS cells were treated for 72 h with indicated talazoparib, temozolomide and SN-38 concentrations. Cell viability was assessed by a MTT assay and is expressed as percentage of untreated cells. Data are shown as mean±s.d. of three independent experiments, each performed in triplicates. MTT, 3-(4,5-dimethylthiazol-2-yl)-2,5-diphenyltetrazolium bromide.

Currently there is no available OS cell line, which is fully deficient in *BRCA1* or *BRCA2*. Although this may constitute a bottleneck for current experimental approaches, some OS cell lines can carry mutations in other genes of the homologous recombination pathway leading to defects functionally analogous to *BRCA1* and *BRCA2* mutations (Fig. 4c). We tested three cell lines, one being a double mutant in *PTEN* and *ATM* (MNNG/HOS) and two with heterozygous SCNA mutations in the checkpoint kinase 2 *CHEK2* (SAOS2) and *FANCD2* gene (SJSA-1), respectively. SJSA-1 and SAOS2 cells were included in the study mainly for comparative purposes since the heterozygous mutations did not seem sufficient to result in homologous recombination repair deficiency. Indeed, *in vitro* tests showed only a limited response of SAOS2 and SJSA cells to a standalone 72-h treatment with the phase-3 poly ADP ribose polymerase (PARP) inhibitor talazoparib (Fig. 5). By contrast, MNNG/HOS cells showed good evidence of IC_50_ after standalone treatment, and even a better response when talazoparib was combined with the alkylating agent temozolomide and the topoisomerase I inhibitor SN-38. The reason for pursuing combined treatment strategies was that the cytotoxicity of talazoparib results from the availability of single-stranded DNA breaks, which temozolomide and SN-38 are particularly effective in creating^34^. Talazoparib concentrations of up to 5 μM used in our experiments have been considered therapeutically achievable concentrations *in vivo* according to a recent study of Hopkins *et al*.^35^.

## Discussion

Cataloguing mutations from 123 tumours has yielded several new insights into the underlying mutational mechanisms in OS. We identified clearly monoclonal mutation events in *TP53* or *RB1* in 47% of cases, whereas additional 40% of tumours could be explained by invoking mutations in alternative driver genes. Eight of these genes—*BRCA2*, *BAP1*, *RET*, *MUTYH*, *ATM*, *PTEN*, *WRN* and *RECQL4*—are well-known Mendelian cancer drivers, whilst the four remaining genes (*ATRX*, *FANCA*, *NUMA1* and *MDC1*) have been reported in the context of cancer susceptibility^2^,^22^,^23^,^24^. In total, 87% of OS could be explained by any of the 14 driver genes. Our findings thus supplement the ‘traditional’ *TP53*-centred model of OS evolution such that molecular pathways functionally similar to *TP53* can drive its initial phases by conferring to genome instability. Tumours without clonal *TP53* mutations may still acquire subclonal *TP53* mutations later which adds ∼25% to the total mutation prevalence. This is important to realize when interpreting findings of other studies as, for example, Chen *et al*.^2^ reported structural and SNV mutations in *TP53* in 30 of 34 genome-sequenced tumours. In a second study, whole-exome sequencing of 53 paediatric OS yielded a 75% prevalence of *TP53* mutations^3^. The difference between these and our estimate might in part result from our inability to identify rearrangements within the first intron of *TP53* (which we estimated at 15% frequency^36^), but other aspects being equal, we conclude that not all previously reported *TP53* mutations are clonal and thus represent the triggering event. Instead we envision a situation, in which multiple oncogenic pathways drive chromosomal aneuploidy and instability in early stages of OS evolution.

**Figure 6:**
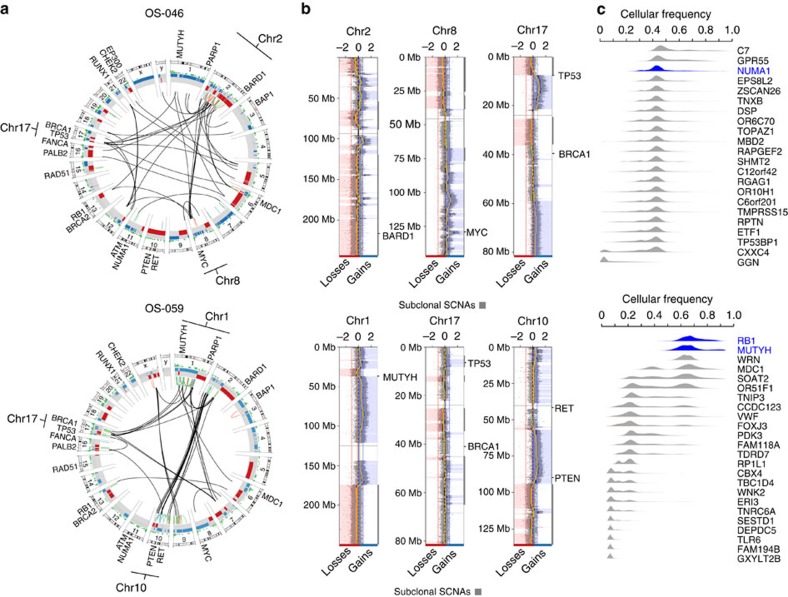
Comprehensive analysis of two tumours. (**a**) A genome-wide plot depicting mutational burden, SCNAs and intra- and inter-chromosomal structural variants. Large structural rearrangements have been detected from whole-genome mate-pair sequencing data as described in Supplementary Note 1 and Supplementary Methods. (**b**) Copy-number profile of selected chromosomes targeted by chromoplexy. Note that chromoplexy likely overlaps with additional structural events including chromothripsis-like events and breakage–bridge fusions. (**c**) Posterior cellular frequencies of somatic exome mutations. Main driver genes are in blue.

Mutational landscapes of two tumours without TP53 mutations (Fig. 6) shall illustrate our hypothesis. The first tumour (OS-046) does likely acquired a homozygous mutation in *NUMA1*, resulting in mitotic segregation errors that at some point led to the chromoplexy of chromosomes 2, 8 and 17 driven by inter-chromosomal exchanges. The evidence supporting *NUMA1* mutation as a triggering event in OS-046 stems from the fact that all somatic SNV mutations were monoclonal, which would be hardly possible if they were sequential to the chromosomal instability. Similarly, an early evolutionary history of the second tumour (OS-059) could have been driven either by a germline *MUTYH* mutation or by a somatic *RB1* mutation. Mutations in both genes were found hemizygous in a tumour, both mutations were clonal events and the functions of both genes are related to chromosomal instability^2^,^20^. However, a parsimonious explanation would still favour a single somatic hit in the *MUTYH* gene over two somatic hits in *RB1*.

In a recent study Nik-Zainal *et al*.^12^ described a distinct profile (which they termed profile D) of substitutions and deletions in breast cancer with *BRCA1/2* mutations, which later was reported by Alexandrov *et al*.^11^ as signature 3 in a pan-cancer analysis. With two outliers (OS-241 and OS-079) we found this signature (as a combination of signatures B and C; Fig. 1b) in 29/31 (94%) tumours from which exome-sequencing data were available. This seems particularly interesting because early evolution of OS has historically been attributed to *TP53* and *RB1* driver mutations, but the root molecular cause of its genomic complexity (in later phases) remains unclear. Seeking to validate this finding we turned to whole-genome copy-number profiles of 123 OS and specifically looked for characteristic large-scale copy-number changes that also constitute the hallmark of *BRCA1/2*-mutated breast cancer^12^. Surprisingly, 84% of the analysed tumours fulfilled these criteria, suggesting that exome findings are likely to be correct and that various degrees of BRCAness are acquired throughout OS evolution. A recent study in ovarian cancer developed a LOH-based homologous recombination deficiency score, which reflects the number of subchromosomal LOH segments with a size exceeding 15 Mbp. Intriguingly, a high homologous recombination deficiency-LOH score, as seen in 78% of OS assayed in our study, was shown to correlate with deficiency in homologous repair and a positive response to a combination treatment including a PARP inhibitor in breast cancer^27^.

Putative deficiency in homologous repair and other similarities between OS and tumours with BRCA-like phenotype indicate a specific weakness that could be therapeutically exploited. Specifically, the inhibition of PARPs contributing to DNA damage repair was shown to induce cell cycle arrest and apoptosis in *BRCA1*-, *BRCA2*- and *PALB2*-deficient breast cancers^29^,^37^. Furthermore, cancer cells with mutations in the *ATM* pathway members and *PTEN* gene were also shown to be sensitive to PARP inhibitors, thus raising the possibility that a ‘synthetic lethality’ approach could also be effective in OS^30^,^33^.

We sought to test this possibility on selected OS cell lines (MNNG/HOS, SAOS2, SJSA-1, ZK58, HOS, MG-63 and U2-OS) but none of them carried bi-allelic BRCA1/2 mutations. We found, however, a good response of MNNG/HOS cells carrying a disruptive gain in the *PTEN* gene and a deletion of the *ATM* gene to a standalone treatment with the phase-3 PARP inhibitor talazoparib. Combined with the alkylating agent temozolomide or the topoisomerase I inhibitor SN-38, 0.1 and 5 μM concentrations of talazoparib led to an even greater decrease of MNNG/HOS cell viability, respectively. Furthermore, a good response of HOS and MG-63 cells to the phase-2 PARP inhibitor olaparip was recently reported by Smith *et al*.^29^.

Using OS cell line experiments impose a few limitations to our study, however. Perhaps the most striking one stems from the paucity of OS cell lines truly deficient in BRCA1/2. This may be surprising, but it should be noted that OS cell lines arise through monoclonal expansions of one cancer cell surviving immortalization, which may not necessarily be the most common cell type in the tumour it is derived from. The molecular portrait of OS presented here prerequisites the existence of intratumour heterogeneity, which is in fact lost *in vitro*. The polyclonal nature of OS also implies that BRCA-like traits of OS genomes might result not only from *BRCA1/2* mutations alone but also from mutations in other genes of the homologous recombination pathway, each of which confers to additional biological properties. These limitations do not invalidate our findings or conclusions, but suggest that, for example, responsiveness of individual tumours to PARP inhibitors will not only depend on the proportion of cancer cells with BRCA-like features but also on the specific mutations and genes that bring about homologous recombination deficiency.

In conclusion, the presented data support a BRCA-like phenotype as a unifying trait of OS independently of which oncogenic pathway drives tumorigenesis. We have shown that at least 14 different genes underlie the disease, including Mendelian cancer drivers for which OS has not been reported to belong within their phenotypic repertoire. More importantly, the effect of mutations within different genes and pathways seem to complement each other and result in a specific signature characteristic for BRCA1/2-deficient tumours. Our findings warrant further testing of PARP inhibitors in experimental settings and eventually sequencing of individual tumours for therapeutically targetable driver gene mutations.

## Methods

### Sample description

The discovery set comprised 31 previously untreated OS samples, paired with peripheral blood or normal tissue. A paired primary tumour and a metastasis was sequenced in one patient. Informed consent was obtained from all patients and ethical approval was obtained from the local ethical committee of Heidelberg (project no. S-327/2011) for the analysis of anonymized samples and for the purpose of driver gene discovery. All tumour samples were re-evaluated by an experienced bone pathologist and confirmed the diagnosis and a tumour content >70% per sample. Genomic DNA was extracted from each tumour and paired blood sample using standard methods. The replication set comprised 92 unpaired frozen pre-therapeutic tumour biopsies, which were subjected to the DNA extraction the same way as the 31 tumours of the discovery set.

### Exome sequencing

Exome capture was performed using the Agilent SureSelect kits (version 4). Samples were quantified using the Qubit system (Invitrogen) and sequencing libraries constructed from 1 μg DNA. Samples were sequenced using the Illumina HiSeq 2000 platform as paired 100-bp reads with Chemistry version 3.0, with the aim of target coverage of 100 × for the blood DNA and 200 × for the tumours. After removal of PCR duplicates using Picard, reads were mapped with Stampy version 1.0.12 onto the Human Reference Genome (GRCh37d5/hg19). SNVs and small indels were called with Platypus version 0.5 using the tumour-normal pairs of bam files together to ensure comparable calls at every locus. Variants were only called if they were assigned sufficiently high posterior probability (phred score >20). We removed the allele bias filter to increase sensitivity. Finally, for selected variants, we made sure that the automatic call matched the data by expert visual inspection of the mapped reads onto the reference genome using read direction colouring on top of the standard integrated genomic viewer scheme.

Annotation was performed using ANNOVAR using hg19 reference genome and 2014 versions of standard databases and functional prediction programs. We excluded duplicated genomic regions (>90% homology) from the analysis and variants within regions with low mapability scores. Variants were annotated with ANNOVAR Refseq gene model using dbSNP(135); 1,000 genomes project allele frequencies (October 2014), 6,500 exome-sequencing project allele frequencies, University of Santa Cruiz (UCSC) segmental duplication scores and UCSC 46 species conservation scores and predictions of functional importance from SIFT, PolyPhen2 and Mutation Taster.

For filtering of variant calls for analysis, calls were first compared between matched constitutional and tumour samples to identify somatic mutations. For the analysis of mutation burden and mutation spectra, we applied the following exclusion filters to somatic variants: (i) presence in a segmental duplication region or a region with mapability score <0.5; (ii) variant present in any read from paired normal sample; (iii) fewer than 10 reads in total at the variant site in the normal sample; (iv) fewer than 8 reads in total in the tumour; (v) fewer 3 three variants in the tumour; variant allele frequency <5% in the tumour; and (vi) presence of variant in public databases (Exome Variant Server, 1,000 genomes project and Complete Genomics 69 reference genomes) at a frequency >2%. Variants identified in constitutional DNA from any of the other local, non-cancer sequencing project (for example, 29 million variants across 284 samples from the Oxford-Illumina WGS500 consortium) were discarded as being more likely due to systematic error in our pipeline than genuine somatic mutations.

### Ion Torrent technical replication

Technical replication of mutations identified by exome sequencing of 31 OS was carried out by Ion Torrent sequencing using a custom Ion AmpliSeq 388 gene panel (gene list and genomic coordinates available as Supplementary Data 2). Approximately 100 ng DNA was used for sequencing. We sequenced to an average depth of 300 × and used the Ion Reporter software for variant calling. Variant calls were annotated using the ANNOVAR software and the same databases as for exome-sequencing data. Variants present in the exomes were assessed alongside the equivalent Ion Torrent data. In addition, visual inspection with the integrated genomic viewer genome browser was required for variant calls with posterior quality scores of 20–30. For specific *RET* mutations we tested additional family members by Sanger sequencing. In addition, we used the Ion AmpliSeq 388 gene panel to validate mutations from 15 randomly picked samples that underwent Illumina MiSeq sequencing.

### Targeted Illumina re-sequencing

Biological replication was carried out using 1 μg of DNA from 92 additional tumours and Illumina MiSeq re-sequencing to an average depth of 480 ×. The custom Nimblegen target enrichment kit was used to capture all exons of 30 genes (that is, 13 main OS drivers and their 17 interaction partners; Supplementary Data 2) and a ∼20-kb region encompassing the *TP53* gene. An average coverage of the target region was 92%. Read mapping, filtering and variant calling was done with the Stampy-Platypus pipeline using the same setting as for the discovery set of 31 exomes.

### Mutation spectra and signatures

For each tumour, somatic SNV frequencies were calculated and the deviation of their spectra from the background (determined across all samples) was assessed using *χ*^2^-test (df=5). The trinucleotide spectrum was normalized according to 3-mer frequencies in the reference exome (Agilent SureSelect (version 4)). Somatic mutation signatures were inferred using the R package^38^, in which a mutation spectrum was decomposed with a non-negative matrix factorization algorithm. The decomposition was performed for prior sets of 2, 3 and 4. The optimal number of signatures (*r*=3) was manually chosen based on the maximum differentiation between the signatures. The signature analysis was repeated 10 times with the same results obtained after each run.

### Affymetrix CytoscanHD array data analysis

Affymetrix CytoscanHD arrays were processed with ChaS 2.1 and Nexus 7.5 software such that SCNA events larger than 50 kb with a minimum support of 21 probes were considered for subsequent analysis. Gene annotations were extracted using Ensembl REST API GRCh37 assembly. We assessed statistical significance of called SCNA regions by an algorithm similar to the GISTIC^39^ analysis, which identifies SCNA with frequency higher than expected by chance. Concretely, for each chromosome SCNA events were resampled to generate uniform distribution and then significance was assessed across all positions in 1-kb windows by deriving Benjamini–Hochberg adjusted *P* values (*q* values). Each candidate SCNA was subjected to survival analysis with subsequent multiple testing correction using Benjamini–Hochberg *q* values <0.05. For event-free survival and overall survival we used Cox proportional hazards regression model.

### *In vitro* cell line experiments

OS cell lines were grown in RPMI 1640 medium (SJSA-1), minimum essential medium supplemented with 10% fetal calf serum (MNNG/HOS) or in McCoy’s 5A medium supplemented with 15% fetal calf serum. Talazoparib was obtained from Selleckchem (Munich, Germany). Control tests for mycoplasm contamination were carried out routinely (two times a month) using a PCR-based and commercially available detection kit according to the manufacturer’s protocol (VenorGem, Minerva Biolabs, Berlin, Germany). Furthermore, authentication of cell lines has been carried out by genotyping using microsatellite markers (Supplementary Data 8) to exclude cross-contaminations. Cell viability was assessed by a 3-(4,5-dimethylthiazol-2-yl)-2,5-diphenyltetrazolium bromide assay according to the manufacturer’s instructions (Roche Diagnostics, Mannheim, Germany). Statistical significance was assessed by Student’s *t*-test (two-tailed distribution, two sample, unequal variance).

## Additional information

**Accession codes:** Exome-sequencing data for osteosarcoma samples have been deposited in the European Nucleotide Archive under the study accession number: PRJEB11430 and the secondary study accession number: ERP012816. Affymetrix CytoscanHD data are available in the ArrayExpress database (www.ebi.ac.uk/arrayexpress) under accession number E-MTAB-3998.

**How to cite this article:** Kovac, M. *et al*. Exome sequencing of osteosarcoma reveals mutation signatures reminiscent of BRCA deficiency. *Nat. Commun.* 6:8940 doi: 10.1038/ncomms9940 (2015).

## Supplementary Material

Supplementary Information Supplementary Figures 1-9, Supplementary Table 1, Supplementary Note 1, Supplementary Methods and Supplementary References

Supplementary Data 1 Mutations in top somatically mutated genes in 31 exomes

Supplementary Data 2 Gene set used for technical replication.

Supplementary Data 3 Thirty genes shortlisted for biological replication.

Supplementary Data 4 Intogen pathway analysis.

Supplementary Data 5 Focal amplifications and deletions in 123 osteosarcomas.

Supplementary Data 6 Significantly over-represented copy-number changes in 123 osteosarcoma genomes.

Supplementary Data 7 The frequency of copy-number changes in BRCA1, BRCA2 and 67 binding partners.

Supplementary Data 8 Short tandem repeat profiles of 8 osteosarcoma cell lines.

## Figures and Tables

**Table 1:** Clinicopathological and summary mutation data for each OS.

ID	Age	Histology	Site	SNV	Indels	Dn:Ds	Ts:Tv	%SCNA	Clonality
OS-046	10	Mixed	Femur	91	24	2.8	1.76	67.45	3
OS-059	13	Osteoblastic	Fibula	50	29	1.18	1.38	67.1	4
OS-061	12	Chondroblastic	Femur	106	36	1.8	1.65	67.5	7
OS-063	17	Teleangiectatic	Humerus	127	48	1.13	1.75	64.3	6
OS-065	15	Osteoblastic	Tibia	78	34	1.75	1.43	44.2	3
OS-079	45	Osteoblastic	Femur	48	20	2.35	1.17	56.2	2
OS-224	11	Osteoblastic	Tibia	310	397	1.62	1.64	57.1	14
OS-225	14	Osteoblastic	Tibia	38	25	1	1.53	50.8	2
OS-226	11	Osteoblastic	Tibia	52	36	1.77	1.36	57.8	6
OS-227	19	Osteoblastic	Femur	29	32	3	1.41	0.2	3
OS-228	13	Chondroblastic	Femur	48	24	2	1.28	59.3	4
OS-230	18	Chondroblastic	Tibia	192	185	1.5	1.23	62.5	11
OS-231	13	Chondroblastic	Tibia	195	246	1.39	1.18	81.8	8
OS-232	21	Extraosseous	Femur	606	529	2.59	1.18	61.1	16
OS-234	17	Osteoblastic	Humerus	46	20	3.8	1.29	60.5	4
OS-235	14	Osteoblastic	Tibia	52	23	1.7	0.79	76.2	5
OS-236	19	Chondroblastic	Femur	46	37	2.6	1.29	59.4	5
OS-237	14	Chondroblastic	Femur	29	28	1.33	1.89	44.9	5
OS-238	17	Osteoblastic	Tibia	63	33	2	2.49	38.3	6
OS-240	15	Osteoblastic	Femur	32	19	3.75	1.66	15.6	6
OS-241	5	Osteoblastic	Fibula	7	19	–	0.4	NA	2
OS-242	14	Osteoblastic	Tibia	35	17	7.33	0.94	26.4	4
OS-250	14	Osteoblastic	Femur	70	35	1.25	0.79	20.5	4
OS-251	9	Osteoblastic	Femur	106	38	1.64	0.89	0.2	9
OS-252	16	Chondroblastic	Femur	253	33	0.82	2.37	34.7	4
OS-253	6	Chondroblastic	Tibia	71	47	3.33	0.82	56.7	3
OS-253M	6	Chondroblastic	Lung	167	56	2.15	0.77	70.1	12
OS-254	27	Mixed	Fibula	33	20	1.25	2.14	30.1	1
OS-255	12	Chondroblastic	Tibia	3,153	459	2.56	2.01	33.9	7
OS-256	17	Osteoblastic	Tibia	50	44	2	1.78	55.6	4
OS-257	12	Mixed	Tibia	1,993	345	1.35	1.92	17.6	4
OS-258	20	Unknown	Humerus	104	29	2.22	1.66	29.4	7

NA, not applicable

SNV denotes high-quality somatic single-nucleotide variants; Dn:Ds denotes the ratio of non-synonymous to synonymous exonic mutations; Ts:Tv denotes a ratio of somatic exonic transitions to transversions; % genome changed by SCNA, per cent of genome affected by copy-number changes; clonality denotes a number of clones estimated by PyClone algorithm.
