# Supplementary material for: Exome sequencing of osteosarcoma reveals mutation signatures reminiscent of BRCA deficiency
Source: Nat Commun. 2015 Dec 3;6:8940. doi: 10.1038/ncomms9940 (PMC4686819; doi:10.1038/ncomms9940)
Supplement: Supplementary Information — Supplementary Figures 1-9, Supplementary Table 1, Supplementary Note 1, Supplementary Methods and Supplementary References [file ncomms9940-s1.pdf]

Supplementary Information

Supplementary Figure 1: The proportion of somatic SNVs in each tumor is shown in a trinucleotide context. The data represent 31 exome-sequenced osteosarcomas. Note that the mutation burden for some cancers was very small.

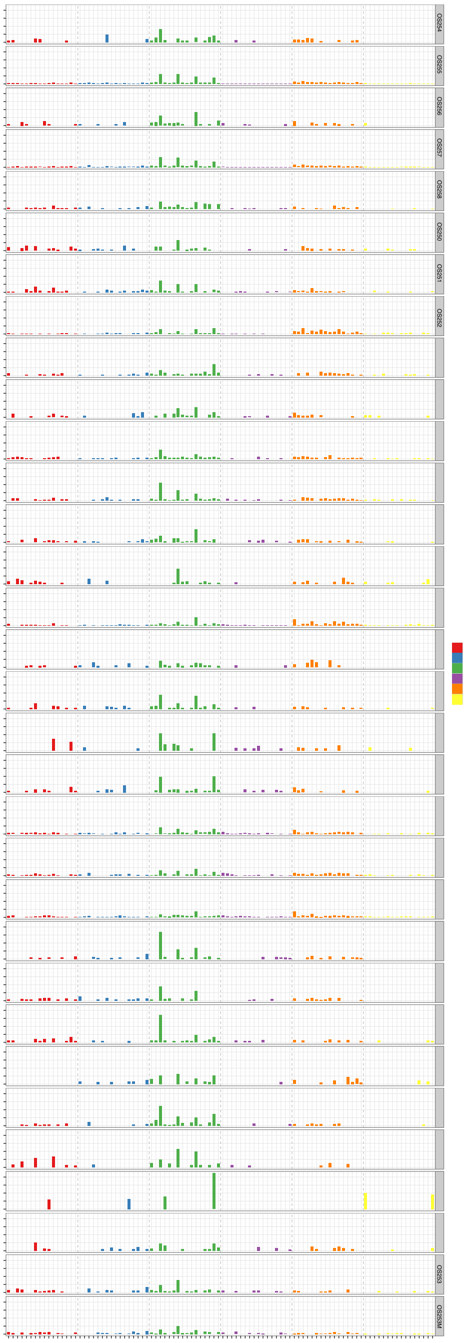

Supplementary Figure 2: Fraction of guanine somatic mutations at CpG dinucleotides.

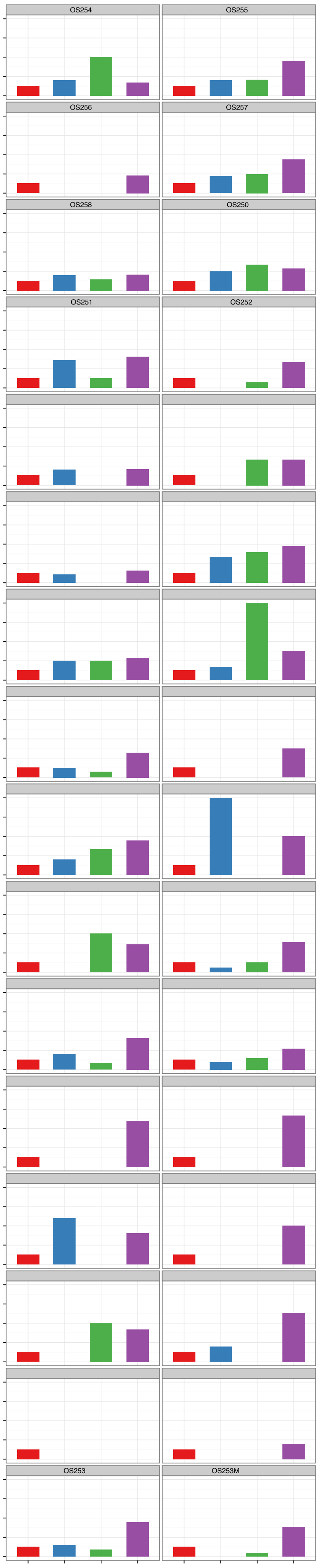

Supplementary Figure 3: Mutation signatures of 31 exome-sequenced osteosarcomas. The top panel shows the contribution of each signature to filtered SNV mutation burden of each tumor, whilst the bottom panel depicts mutations within a trinucleotide context split by a signature.

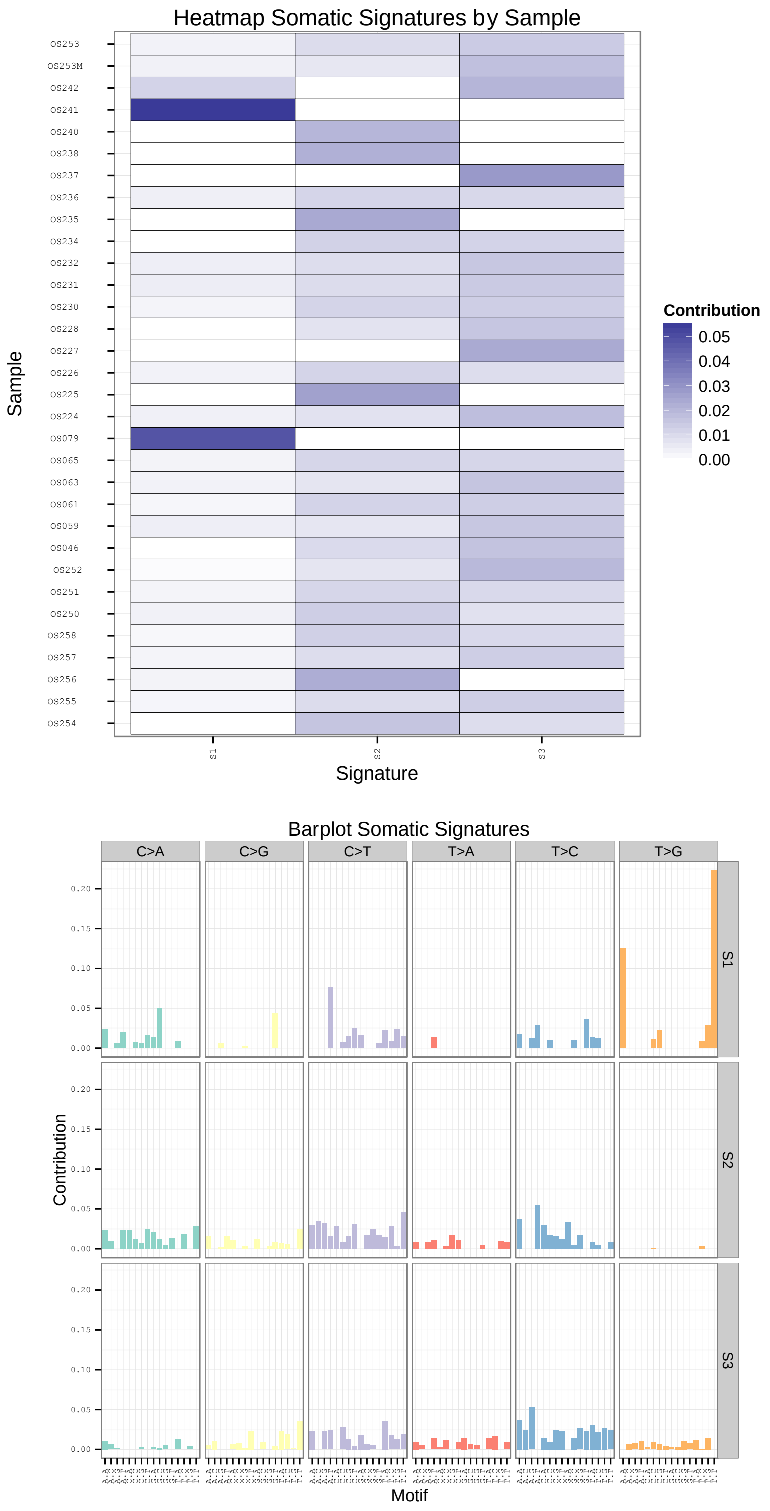

Supplementary Figure 4: Regions enriched for copy-number-loss breakpoints within three osteosarcoma driver genes (FANCA, ATRX and RET).

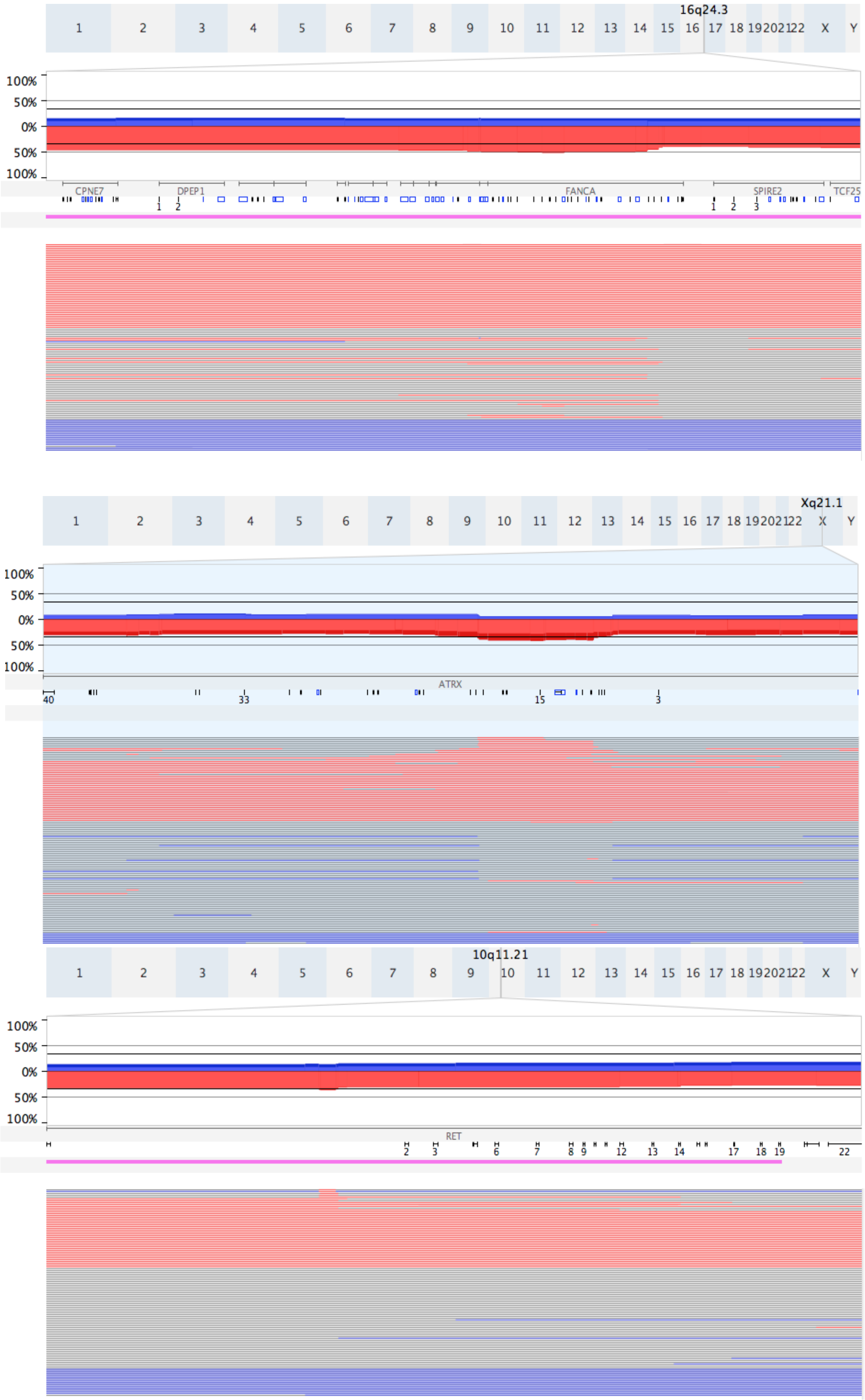

Supplementary Figure 5: Focal amplification and deletions in osteosarcoma. The SCNA profile of 123 tumors is shown on the top, followed by a heat map representation of individual copy-number profiles (bottom). Focal SCNA events with frequency >15% are listed in the Supplementary Data 5.

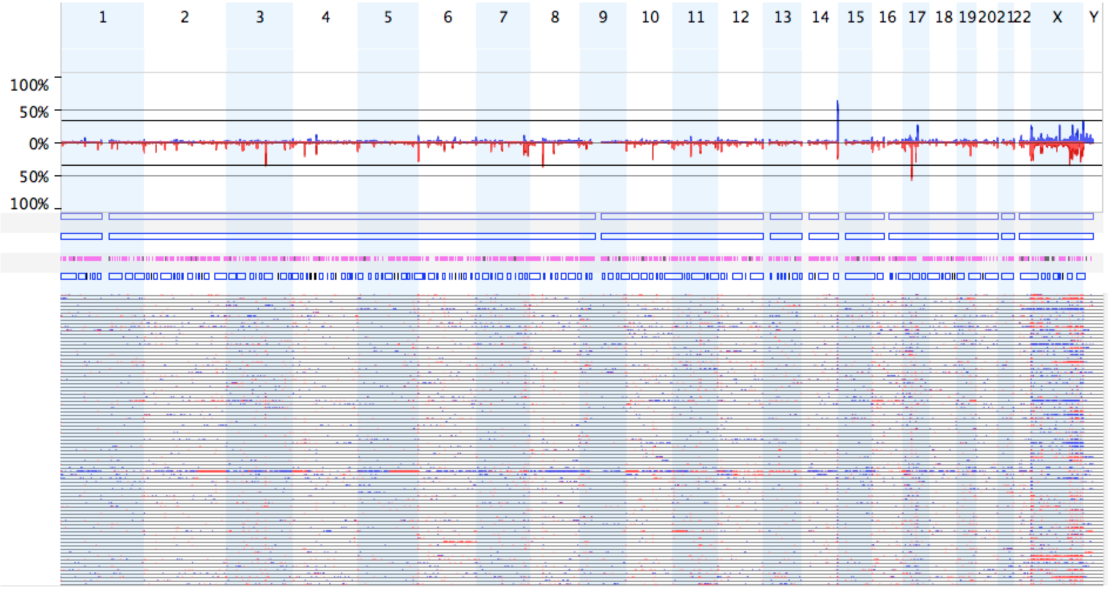

Supplementary Figure 6: The algorithm used for the identification of over-represented SCNA events in osteosarcoma. We used random resampling of SCNA and subsequently tested for statistical significance of short chromosomal regions by using Binomial distribution.

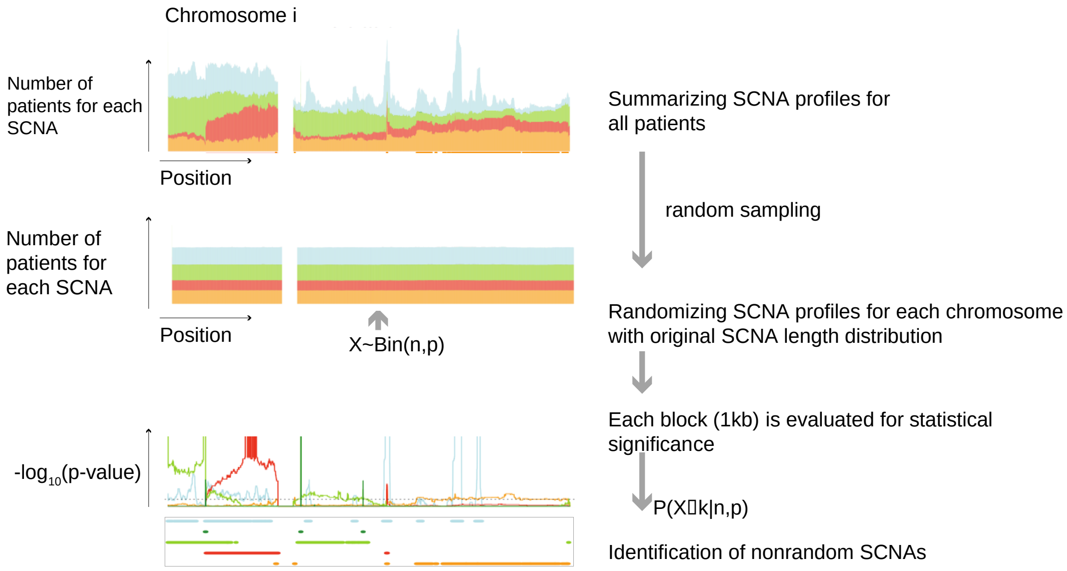

Supplementary Figure 7: Two distinct types of osteosarcoma genomes identified by clustering of SCNA profiles. HZ: homozygous, Amp: amplification, CN: copy-number.

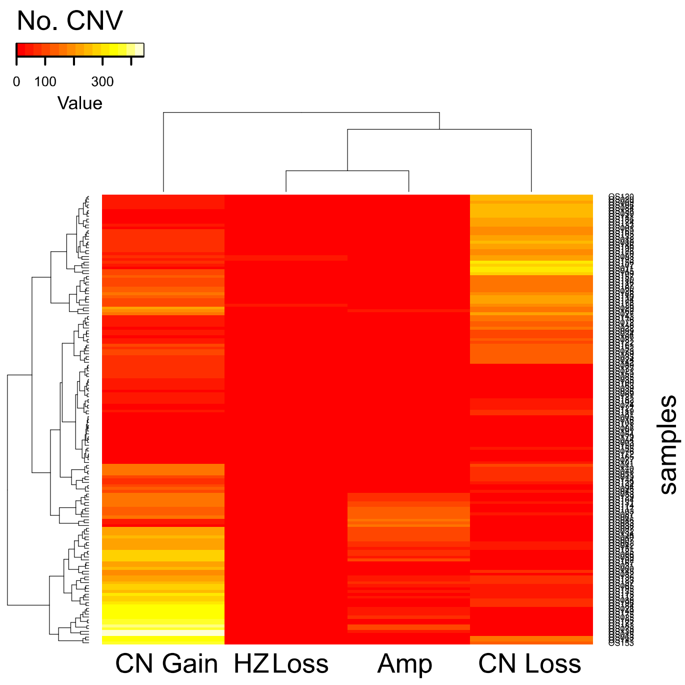

Supplementary Figure 8: The combined (I) and the individual (II) effect of SCNA events on the survival of respective patients. The P value has been calculated using Cox hazard regression model.

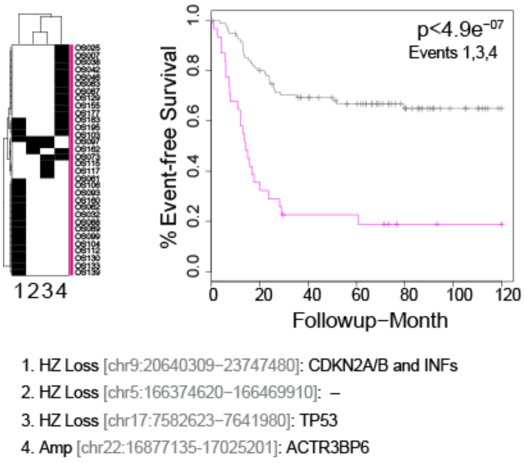

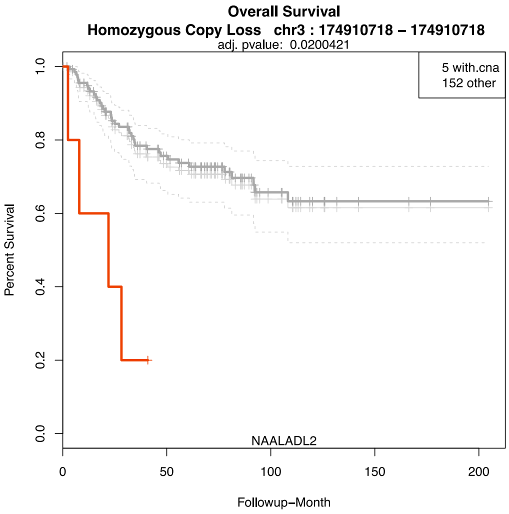
Overall Survival
Homozygous Copy Loss chr3 : 174910718 – 174910718
adj. pvalue: 0.0200421
5 with.cna
152 other
NAALADL2
Percent Survival
Followup–Month

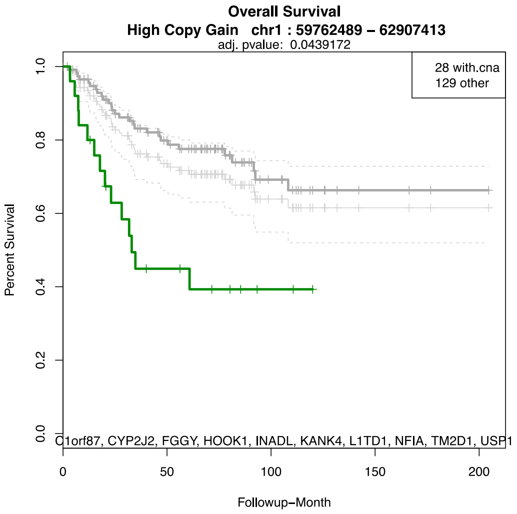
Overall Survival
High Copy Gain chr1 : 59762489 – 62907413
adj. pvalue: 0.0439172
28 with.cna
129 other
C1orf87, CYP2J2, FGGY, HOOK1, INADL, KANK4, L1TD1, NFIA, TM2D1, USP1
Percent Survival
Followup–Month

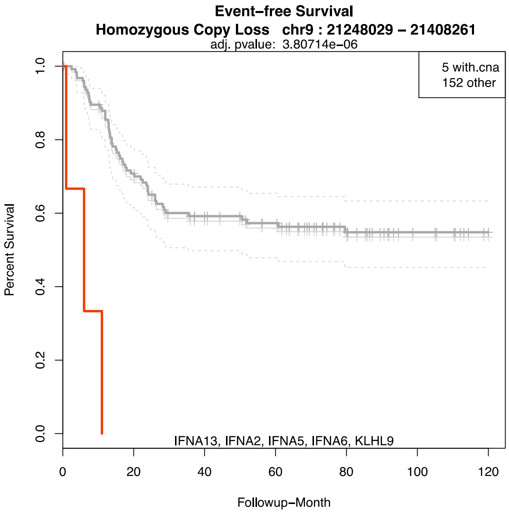
Event–free Survival
Homozygous Copy Loss chr9 : 21248029 – 21408261
adj. pvalue: 3.80714e–06
5 with.cna
152 other
IFNA13, IFNA2, IFNA5, IFNA6, KLHL9
Percent Survival
Followup–Month

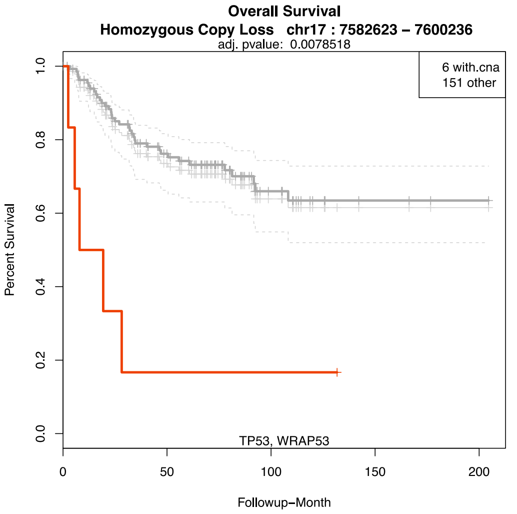
Overall Survival
Homozygous Copy Loss chr17 : 7582623 – 7600236
adj. pvalue: 0.0078518
6 with.cna
151 other
TP53, WRAP53
Percent Survival
Followup–Month

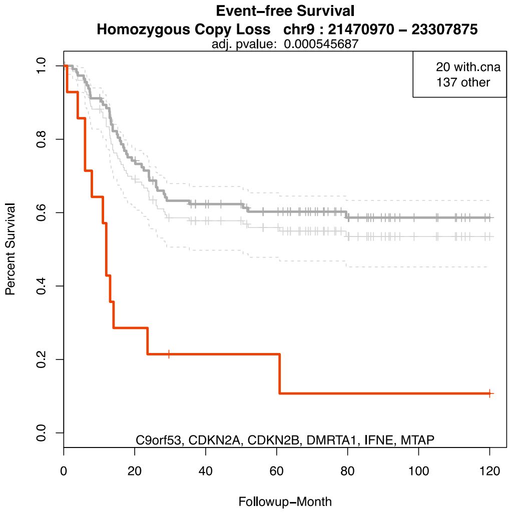
Event–free Survival
Homozygous Copy Loss chr9 : 21470970 – 23307875
adj. pvalue: 0.000545687
20 with.cna
137 other
C9orf53, CDKN2A, CDKN2B, DMRTA1, IFNE, MTAP
Percent Survival
Followup–Month

Supplementary Figure 9: The effect of 3 SCNA loci on the age of onset.

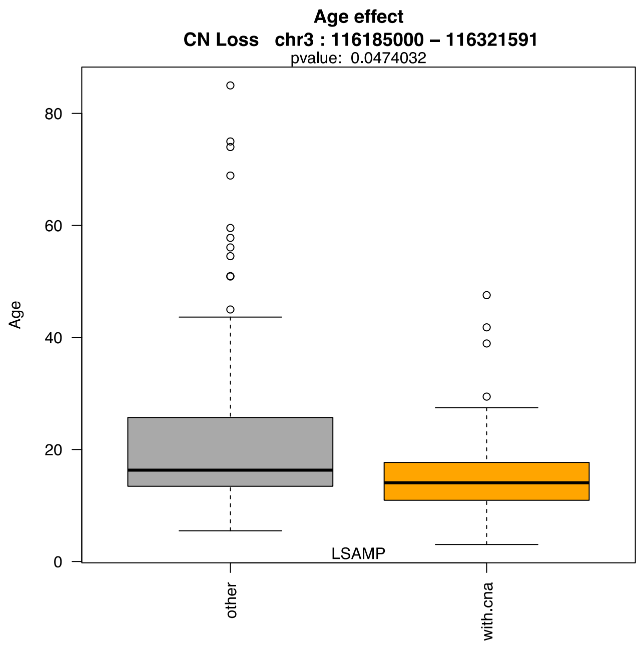

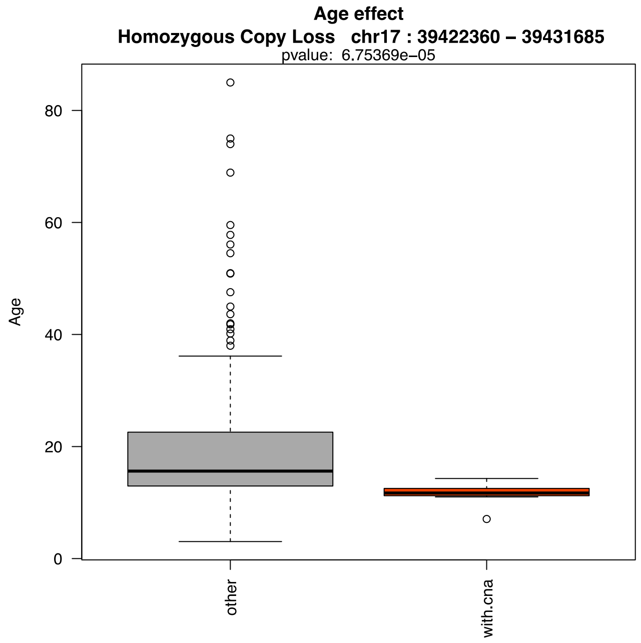

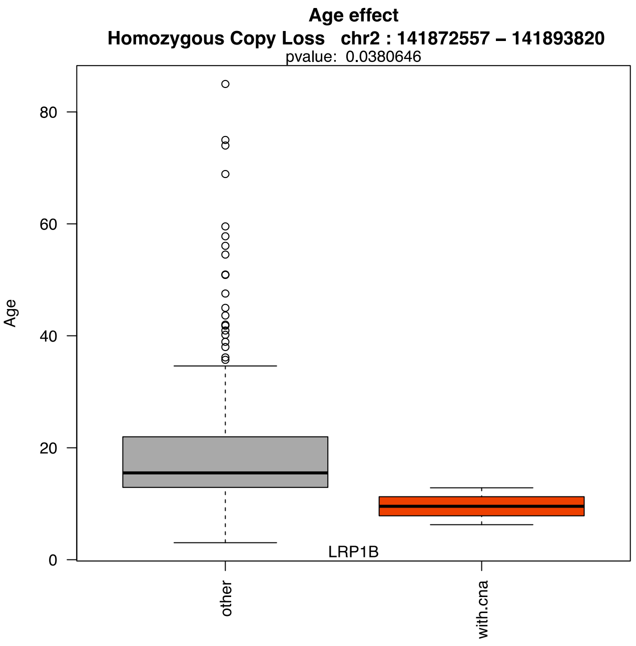
Age effect
Homozygous Copy Loss chr2 : 141872557 – 141893820
pvalue: 0.0380646
Age
80
60
40
20
0
LRP1B
other
with.cna

Supplementary Table 1

Osteosarcoma driver mutations and mutations in genes fulfilling the criteria for further analysis

Genomic Position	Ref	Alt	VAF	dbSNP ID	Mutation Type	Somatic Status	Sample ID	Gene	DNA Change	SIFT, PP2, Gerp++	Protein Change	Prosite ProRule ID
chr1:35656446	C	A	0.35	-	Missense	Unknown	OS-049	SFPQ	c.G1168T	0.19, 0.96, 5.67	p.A390S	PRU00176
chr1:45795084	G	A	0.75	rs140118273	Missense	Germline	OS-059	MUTYH	c.C1502T	0.04, 0.91, 0.17	p.S501F	
chr1:45797135	C	-	0.17	-	Frameshift deletion	Unknown	OS-188	MUTYH	c.1238delG	NA, NA, NA	p.W413fs	PRU00794
chr1:45797157	G	T	0.56	rs144079536	Missense	Unknown	OS-038	MUTYH	c.C1216A	0.12, 0.99, 4.50	p.L406M	PRU00794
chr1:45798350	C	A	0.60	-	Stop gain	Unknown	OS-015	MUTYH	c.G544T	0.12, NA, 4.52	p.E182X	
chr1:45798475	T	C	0.80	rs34612342	Missense	Germline	OS-252	MUTYH	c.A494G	0.00, 1.00, 5.01	p.Y165C	
chr1:45798475	T	C	0.28	rs34612342	Missense	Germline	OS-106	MUTYH	c.A494G	0.00, 1.00, 5.01	p.Y165C	
chr1:45798624	G	A	0.20	-	Missense	Unknown	OS-102	MUTYH	c.C428T	0.00, 1.00, 5.15	p.P143L	
chr6:30670966	A	G	0.17	-	Missense	Unknown	OS-034	MDC1	c.T5780C	0.00, 1.00, 5.32	p.V1927A	PRU00033
chr6:30671011	A	T	0.13	-	Missense	Unknown	OS-115	MDC1	c.T5735A	0.00, 1.00, 5.32	p.L1912Q	PRU00033
chr6:30680124	C	T	0.77	rs139338660	Missense	Unknown	OS-088	MDC1	c.G1595A	0.02, 0.96, 1.89	p.G532E	
chr6:30681838	G	A	0.40	-	Missense	Unknown	OS-129	MDC1	c.C259T	0.09, 1.00, 4.75	p.P87S	PRU00086
chr8:30954313	C	A	0.11	-	Missense	Somatic	OS-063	WRN	c.C1928A	0.00, 1.00, 5.94	p.P643Q	PRU00541
chr8:31012237	C	G	0.91	rs78488552	Missense	Germline	OS-230	WRN	c.C3785G	0.00, 0.99, 5.48	p.T1262R	
chr8:31014943	AGCGGTGAAAGCTGG	-	0.19		In-frame deletion	Somatic	OS-061	WRN	c.3879_3893del	NA, NA, NA	p.1293_1298del	
chr8:145737373	C	T	0.49	rs36078464	Missense	Germline	OS-227	RECQL4	c.G3314A	NA, NA, NA	p.G1105D	
chr8:145738666	G	A	0.25		Stop gain	Somatic	OS-077	RECQL4	c.C2398T	NA, NA, NA	p.Q800X	PRU00542
chr8:145738669	C	T	0.38	rs34293591	Missense	Germline	OS-238	RECQL4	c.G2395A	NA, NA, NA	c.G2395A	
chr10:43600398	A	G	0.16	rs145633958	Splice site	Unknown	OS-073	RET	c.626-2A>G	NA, NA, 4.79	Splicing	
chr10:43601917	G	A	0.75		Missense	Germline	OS-224	RET	c.G961A	0.3, 0.12, -1.32	p.G321R	
chr10:43604580	C	T	0.86		Missense	Unknown	OS-128	RET	c.C1165T	0.01, 0.92, 1.05	p.L389F	
chr10:43609994	C	T	0.42	rs148935214	Missense	Germline	OS-250	RET	c.C1946T	0.01, 1.00, 4.34	p.S649L	
chr10:43612152	A	G	0.15		Missense	Unknown	OS-011	RET	c.A2257G	0.01, 0.99, 5.65	p.T753A	PRU00159
chr10:43622095	A	G	0.47		Missense	Germline	OS-242	RET	c.A3112G	0.01, 0.98, 5.09	p.T1038A	
chr10:89720715	A	G	0.11		Missense	Unknown	OS-024	PTEN	c.A866G	NA, 0.98, 5.13	p.K289R	PRU00589
chr11:71715733	G	A	0.39	rs35681270	Missense	Unknown	OS-115	NUMA1	c.C5959T	0.00, 1.00, 4.73	p.R1987C	
chr11:71717155	A	G	0.11		Missense	Unknown	OS-148	NUMA1	c.T5618C	0.00, 1.00, 5.11	p.L1873S	
chr11:71720030	G	A	0.48	rs74985106	Missense	Germline	OS-046	NUMA1	c.C5041T	0.01, 1.00, 5.56	p. R1681C	
chr11:108098563	C	T	0.45	rs3218684	Missense	Germline	OS-252	ATM	c.C133T	0.00, 0.96, 3.16	p.R45W	
chr11:108164059	A	C	0.43		Missense	Germline	OS-237	ATM	c.A4631C	0.04, 0.88, 2.90	p.Y1544S	
chr11:108175463	A	T	0.70	rs1801673	Missense	Germline	OS-224	ATM	c.A5558T	0.02, 0.88, 5.52	p.D1853V	
chr13:32972626	A	T	0.45	rs11571833	Stop gain	Germline	OS-254	BRCA2	c.A9976T	NA, NA, 0.16	p.K3326X	
chr13:48934198	T	A	0.58		Stop gain	Somatic	OS-242	RB1	c.T653A	NA, NA, 5.59	p.L218X	
chr13:48936983	C	T	0.59		Stop gain	Unknown	OS-151	RB1	c.C751T	NA, NA, 5.11	p.R251X	
chr13:48936995	C	T	0.81		Stop gain	Unknown	OS-015	RB1	c.C763T	NA, NA, 3.11	p.R255X	
chr13:48936995	C	T	0.79		Stop gain	Somatic	OS-061	RB1	c.C763T	NA, NA, 3.11	p.R255X	

chr13:48939028	A	T	0.82		Splice site	Somatic	OS-059	RB1	c.862-2A>T	NA, NA, 5.36	Splicing	
chr13:48954302	A	G	0.11		Missense	Unknown	OS-080	RB1	c.A1423G	0.06, 0.98, -2.73	p.K475E	
chr13:48955381	A	G	0.92		Splice site	Unknown	OS-026	RB1	c.1499-2A>G	NA, NA, 5.34	Splicing	
chr13:48955580	G	A	0.19		Splice site	Unknown	OS-027	RB1	c.1695+1G>A	NA, NA, 5.34	Splicing	
chr13:49030486	G	-	0.66		Splice site	Unknown	OS-007	RB1	c.1960+1G>-	NA, NA, NA	Splicing	
chr13:49037972	G	A	0.52		Splice site	Somatic	OS-254	RB1	c.2211+1G>A	NA, NA, 6.11	Splicing	

Genomic Position	Ref	Alt	VAF	dbSNP ID	Mutation Type	Somatic Status	Sample ID	Gene	DNA Change	SIFT, PP2, Gerp++	Protein Change	Prosite ProRule ID
chr16:89811418	G	A	0.91		Missense	Germline	OS-226	FANCA	c.C3575T	0.02, 0.92, -0.39	p.P1192L	
chr16:89813075	G	A	0.83	rs143671872	Missense	Unknown	OS-053	FANCA	c.C3430T	0.01, 0.99, 3.20	p.R1144W	
chr16:89871756	A	G	0.13		Missense	Unknown	OS-026	FANCA	c.T641C	0.00, 1.00, 2.70	p.L214P	
chr17:7578527	A	G	0.71		Missense	Unknown	OS-103	TP53	c.T403C	0.00, 1.00, 5.48	p.C135R	
chr17:7578525	G	C	0.93		Missense	Unknown	OS-026	TP53	c.C405G	0.01, 1.00, 3.50	p.C135W	
chr17:7578461	C	A	0.62	rs121912654	Missense	Unknown	OS-136	TP53	c.G469T	0.01, 1.00, 2.42	p.V157F	
chr17:7578431	G	A	0.77		Stop gain	Unknown	OS-186	TP53	c.C499T	NA, NA, 4.63	p.Q167X	
chr17:7578272	G	A	0.22		Missense	Unknown	OS-084	TP53	c.C577T	0.00, 1.00, 5.41	p.H193Y	
chr17:7578212	G	A	0.77		Stop gain	Unknown	OS-072	TP53	c.C637T	NA, NA, 3.52	p.R213X	
chr17:7578190	T	C	0.40		Missense	Unknown	OS-161	TP53	c.A659G	0.00, 1.00, 5.28	p.Y220C	
chr17:7577598	TCAG	-	0.54		Frameshift deletion	Unknown	OS-097	TP53	c.680_683del	NA, NA, NA	p.227fs	
chr17:7577574	T	C	0.84		Missense	Unknown	OS-088	TP53	c.A707G	0.00, 1.00, 0.53	p.Y236C	
chr17:7577539	G	C	0.60		Missense	Unknown	OS-125	TP53	c.C742G	0.00, 1.00, 2.56	p.R248G	
chr17:7577538	C	T	0.80	rs11540652	Missense	Unknown	OS-183	TP53	c.G743A	0.01, 1.00, 3.65	p.R248Q	
chr17:7577538	C	T	0.47	rs11540652	Missense	Unknown	OS-054	TP53	c.G743A	0.01, 1.00, 3.65	p.R248Q	
chr17:7577515	T	G	0.30		Missense	Unknown	OS-140	TP53	c.A766C	0.00, 1.00, 3.51	p.T256P	
chr17:7577511	A	C	0.42		Missense	Unknown	OS-027	TP53	c.T770G	0.00, 1.00, 3.53	p.L257R	
chr17:7577498	C	A	0.35		Splice site	Unknown	OS-015	TP53	c.782+1G>T	NA, NA, 4.31	Splicing	
chr17:7577120	C	T	0.78	rs28934576	Splice site	Unknown	OS-104	TP53	c.G818A	0.01, 1.00, 4.92	p.R273H	
chr17:7577096	T	A	0.47		Missense	Unknown	OS-140	TP53	c.A842T	0.00, 1.00, 5.13	p.D281V	
chr17:7577095	G	C	0.89		Missense	Unknown	OS-034	TP53	c.C843G	0.00, 0.98, 5.13	p.D281E	
chr17:7577095	G	T	0.33		Missense	Unknown	OS-011	TP53	c.C843A	0.00, 0.98, 5.13	p.D281E	
chr17:7577094	G	A	0.45	rs28934574	Missense	Unknown	OS-077	TP53	c.C844T	0.00, 1.00, 1.49	p.R282W	
chr17:7577087	GTGCGCCG	-	0.63		Frameshift	Germline	OS-228	TP53	c.844_851del	NA, NA, NA	p.282fs	
chr17:7574018	G	A	0.41		Missense	Unknown	OS-049	TP53	c.C1009T	0.00, 0.69, 3.38	p.R337C	
chr22:41568501	A	G	0.11		Splice site	Unknown	OS-161	EP300	c.4453-2A>G	NA, NA, 5.96	Splicing	
chr22:41527587	-	CGCGAA	0.13		In-frame insertion	Unknown	OS-191	EP300	c.1478_1479insCGCGAA	NA, NA, NA	p.N493delinsNAN	
chr22:41573745	-	CC	0.16		Frameshift insertion	Somatic	OS-224	EP300	c.6030insCC	NA, NA, NA	p.M2010fs	
chrX:76776897	T	A	0.99		Missense	Unknown	OS-141	ATRX	c.A7055T	0.00, 1.00, 5.06	p.D2352V	

chrX:76778809	A	T	0.62	Missense	Unknown	OS-106	ATRX	c.T6770A	0.00, 1.00, 5.35	p.L2257H	
chrX:76778855	G	A	0.17	Stop gain	Unknown	OS-170	ATRX	c.C6724T	NA, NA, 5.46	p.Q2242X	
chrX:76814238	C	T	0.27	Missense	Unknown	OS-073	ATRX	c.G6406A	0.00, 1.00, 5.35	p.D2136N	PRU00542
chrX:76938341	-	CATA	0.39	Frameshift insertion	Unknown	OS-129	ATRX	c.2406insTATG	NA, NA, NA	p.I803fs	
chrX:76939418	G	A	0.39	Stop gain	Somatic	OS-063	ATRX	c.C1330T	NA, NA, 2.89	p.R444X	
chrX:76940480	T	C	0.13	Missense	Unknown	OS-191	ATRX	c.A613G	0.24, 0.97, 5.54	p.M205V	PRU00865
chrX:76855982	A	-	0.41	Frameshift deletion	Somatic	OS-235	ATRX	c.5618delT	NA, NA, NA	p.F1873fs	
chrX:76891541	C	A	0.78	Stop gain	Somatic	OS-236	ATRX	c.G4564T	NA, NA, NA	p.E1522X	
chrX:76937876	G	-	0.11	Frameshift deletion	Somatic	OS-232	ATRX	c.2872delC	NA, NA, NA	p.Q958fs	
chrX:76938727	GTTACAGG	-	0.24	Frameshift deletion	Somatic	OS-061	ATRX	c.2014_2021del	NA, NA, NA	p.672fs	

Data are from the discovery set of 31 exomes and the replication set of 92 tumors. The table shows selected cancer driver genes with mutations of a moderate or greater predicted functional effect. LOH, loss of heterozygosity.

Supplementary Note 1

The identification of genome rearrangements from mate-pair sequencing data

In seven tumors where additional whole genome mate-pair sequencing data were available, we annotated and investigated large structural variants based on the presence of split sequencing reads between and within chromosomes. Structural variant calls were cross-referenced with the list of significant SCNA events and loci with chromothripsis-like events, with the latter defined according to Cai *et al.*[1] as chromosome arms with at least 12 copy-number transitions with a $\log_{10}$ likelihood ratio higher than 8. Medians of 118 (cross-sample range 40-194) intra- and 43 (cross-sample range 8-117) inter-chromosomal exchanges were found per genome of mate-pair sequenced tumors. After filtering for events within significantly over-represented SCNA regions only one reciprocal inter-chromosomal 5’ to 3’ translocation between promotor regions of *DIS3L2 (*chr2:232’825’955) and *RB1* (chr13:48’877’887) remained in the list. Specifically, none of the *TP53* intron 1 rearrangements previously reported in OS were detected in the 7 Illumina mate-pair sequenced tumors.

Supplementary Methods

Whole-Genome Mate-Pair Sequencing and Structural Variation Detection

Whole genome sequencing long-range paired-end mapping were performed as previously described[2] and sequenced on Illumina HiSeq sequencers to an average depth of 24x, with the raw length of the reads displaying a median of 101bp and a median insert size of the sequenced libraries of 4,600 bp. Structural variants larger than 500bp were detected using previously-described settings[2] and Delly analytical pipeline[3].

Supplementary References

1. Cai, H. *et al.* Chromothripsis-like patterns are recurring but heterogeneously distributed features in a survey of 22,347 cancer genome screens. *BMC Genomics* **15,** 82 (2014).
2. Weischenfeldt, J. *et al.* Integrative genomic analyses reveal an androgen-driven somatic alteration landscape in early-onset prostate cancer. *Cancer Cell* **23,** 159–170 (2013).
3. Rausch, T. *et al.* DELLY: structural variant discovery by integrated paired-end and split-read analysis. *Bioinformatics* **28,** i333–i339 (2012).
